# 2,4-Dichloro-7,8-dimethyl­quinoline

**DOI:** 10.1107/S1600536810020386

**Published:** 2010-06-05

**Authors:** R. Subashini, F. Nawaz Khan, T. Rajashekar Reddy, Venkatesha R. Hathwar, Mehmet Akkurt

**Affiliations:** a Organic and Medicinal Chemistry Research Laboratory, Organic Chemistry Division, School of Advanced Sciences, VIT University, Vellore 632 014, Tamil Nadu, India; b Solid State and Structural Chemistry Unit, Indian Institute of Science, Bangalore 560 012, Karnataka, India; c Department of Physics, Faculty of Arts and Sciences, Erciyes University, 38039 Kayseri, Turkey

## Abstract

There are two independent mol­ecules in the asymmetric unit of the title compound, C_11_H_9_Cl_2_N, both of which are essentially planar [maximum deviations of 0.072 (5) and 0.072 (7) Å]. In the crystal structure, weak π–π stacking inter­actions [centroid-centroid distances = 3.791 (3) Å and 3.855 (3) Å] link pairs of mol­ecules.

## Related literature

For the properties and applications of related compounds, see: Biavatti *et al.* (2002 ▶); Fournet *et al.* (1981 ▶); McCormick *et al.* (1996 ▶); Towers *et al.* (1981 ▶); Ziegler & Gelfert (1959 ▶). For similar crystal structures, see: Subashini *et al.* (2009 ▶); Somvanshi *et al.* (2008 ▶).
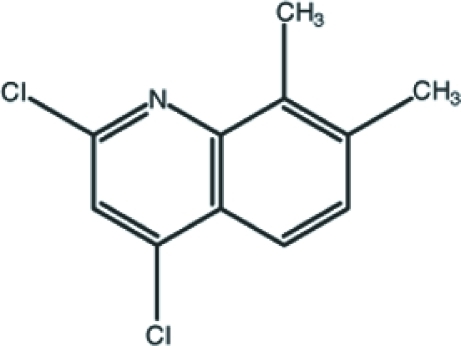


## Experimental

### 

#### Crystal data

C_11_H_9_Cl_2_N
*M* *_r_* = 226.09
Orthorhombic, 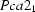

*a* = 20.3054 (9) Å
*b* = 3.9992 (2) Å
*c* = 25.5743 (11) Å
*V* = 2076.77 (17) Å^3^
*Z* = 8
Mo *K*α radiation
μ = 0.58 mm^−1^
*T* = 295 K
0.30 × 0.24 × 0.15 mm

#### Data collection

Oxford Xcalibur Eos (Nova) CCD detector diffractometer
Absorption correction: multi-scan (*CrysAlis PRO RED*; Oxford Diffraction, 2009 ▶) *T*_min_ = 0.845, *T*_max_ = 0.918
19807 measured reflections
4009 independent reflections
2599 reflections with *I* > 2σ(*I*)
*R*_int_ = 0.048

#### Refinement

*R*[*F* ^2^ > 2σ(*F* ^2^)] = 0.049
*wR*(*F* ^2^) = 0.119
*S* = 0.94
4009 reflections
257 parameters
1 restraint
H-atom parameters constrained
Δρ_max_ = 0.32 e Å^−3^
Δρ_min_ = −0.19 e Å^−3^
Absolute structure: Flack (1983 ▶), 1943 Friedel pairs
Flack parameter: 0.15 (10)

### 

Data collection: *CrysAlis PRO CCD* (Oxford Diffraction, 2009 ▶); cell refinement: *CrysAlis PRO CCD*; data reduction: *CrysAlis PRO RED* (Oxford Diffraction, 2009 ▶); program(s) used to solve structure: *SHELXS97* (Sheldrick, 2008 ▶); program(s) used to refine structure: *SHELXL97* (Sheldrick, 2008 ▶); molecular graphics: *ORTEP-3* (Farrugia, 1997 ▶); software used to prepare material for publication: *WinGX* (Farrugia, 1999 ▶).

## Supplementary Material

Crystal structure: contains datablocks global, I. DOI: 10.1107/S1600536810020386/vm2029sup1.cif

Structure factors: contains datablocks I. DOI: 10.1107/S1600536810020386/vm2029Isup2.hkl

Additional supplementary materials:  crystallographic information; 3D view; checkCIF report

## Figures and Tables

**Fig. 1.**
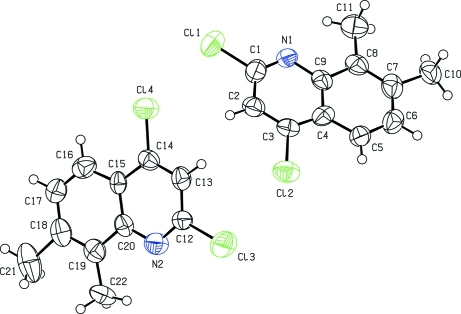
View of the two molecules in the same asymmetric unit of (I), showing the atom-numbering scheme and displacement ellipsoids drawn at the 50% probability level.

**Fig. 2.**
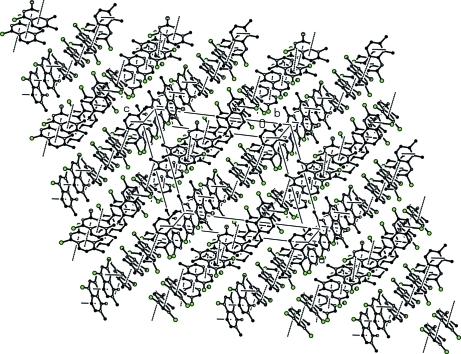
The molecular packing of (I) showing π-π stacking interactions (dashed lines) between the adjacent molecules down b axis. H atoms are omitted for clarity.

## supplementary crystallographic information

### Comment

A wide range of medicinal properties have already been identified for compounds containing the quinoline ring system including antiprotozoal (Fournet *et al.*, 1981), antibacterial (Towers *et al.*, 1981), antifungal (Biavatti *et al.*, 2002) and antiviral activities (McCormick *et al.*, 1996). Reaction of aniline with malonic acid in an excess of phosphorus oxychloride at reflux to give 2,4-dichloroquinoline was first reported by Ziegler & Gelfert (1959). A similar derivative of quinoline was synthesized from the mixture of *p*-toluidine and malonic acid in a one-pot reaction from an aryl amine, malonic acid and phosphorous oxychloride and its cytotoxicity has been reported (Somvanshi *et al.*, 2008). Another derivative of quinoline prepared from *p*-anisidine and phosphorous oxychloride has been reported (Subashini *et al.*, 2009). In continuous of our work, the crystal structure of another derivative is reported in this paper.

The molecules A (Cl1/Cl2/N1/C1–C11) and B (Cl3/Cl4/N2/C12–C22) in the asymmetric unit of the title compound (I) are shown in Fig. 1. In both molecules A and B, the bond lengths and angles are comparable with those of similar structures (Somvanshi *et al.*, 2008; Subashini *et al.*, 2009). The molecules A and B are essentially planar, except the H atoms of their methyl groups, with maximum deviations of 0.072 (5)Å for C10 and 0.072 (7)Å for C21, respectively. Fitting of the non-H atoms of molecules A and B results in an r.m.s. fit of 0.063 Å). The least-squares plane through molecule A makes a dihedral angle of 56.72 (14)° with that of molecule B.

Weak intramolecular C—H···Cl and C—H···N interactions contribute to the stabilization of the molecular conformation of (I) (Table 1). In the crystal structure, weak π-π stacking interactions [*Cg*1···*Cg*2(*x*, 1 + *y*, *z*) = 3.791 (3) Å and *Cg*4···*Cg*5 (*x*, 1 + *y*, *z*) = 3.855 (3) Å; where *Cg*1, *Cg*2, *Cg*4 and *Cg*5 are centroids of the N1/C1–C4/C9, C4–C9, N2/C12–C15/C20 and C15–C20 rings, respectively] link pairs of molecules. In the structure, no classical hydrogen bonds are observed. Fig. 2 shows the crystal packing down the *b* axis.

### Experimental

2,3-Dimethylaniline (10 mmol) and malonic acid (10 mmol) were heated under reflux in phosphorus oxychloride (30 ml), with stirring, for 5 h. The mixture was cooled, poured into crushed ice with vigorous stirring and then made alkaline with 5 *M* sodium hydroxide. Filtration gave the crude product as a brown solid. Column chromatography (95:5 hexane–EtOAc) yielded the pure 2,4-dichloro-7,8-dimethylquinoline. White needles of the synthesized compound have been grown from DMSO.

### Refinement

H atoms were positioned geometrically with C—H = 0.93 and 0.96 Å, for aromatic and methyl H and refined as a riding method, with *U*_iso_(H) = 1.2 or 1.5*U*_eq_(C).

### Crystal data

**Table:**

C_11_H_9_Cl_2_N	*F*(000) = 928
*M**_r_* = 226.09	*D*_x_ = 1.446 Mg m^−^^3^
Orthorhombic, *P**c**a*2_1_	Mo *K*α radiation, λ = 0.71073 Å
Hall symbol: P 2c -2ac	Cell parameters from 895 reflections
*a* = 20.3054 (9) Å	θ = 1.8–24.7°
*b* = 3.9992 (2) Å	µ = 0.58 mm^−^^1^
*c* = 25.5743 (11) Å	*T* = 295 K
*V* = 2076.77 (17) Å^3^	Needle, colourless
*Z* = 8	0.30 × 0.24 × 0.15 mm

### Data collection

**Table:**

Oxford Xcalibur Eos (Nova) CCD detector diffractometer	4009 independent reflections
Radiation source: Enhance (Mo) X-ray Source	2599 reflections with *I* > 2σ(*I*)
graphite	*R*_int_ = 0.048
ω scans	θ_max_ = 26.0°, θ_min_ = 2.6°
Absorption correction: multi-scan (*CrysAlis PRO* RED; Oxford Diffraction, 2009)	*h* = −25→25
*T*_min_ = 0.845, *T*_max_ = 0.918	*k* = −4→4
19807 measured reflections	*l* = −31→31

### Refinement

**Table:**

Refinement on *F*^2^	Secondary atom site location: difference Fourier map
Least-squares matrix: full	Hydrogen site location: inferred from neighbouring sites
*R*[*F*^2^ > 2σ(*F*^2^)] = 0.049	H-atom parameters constrained
*wR*(*F*^2^) = 0.119	*w* = 1/[σ^2^(*F*_o_^2^) + (0.0652*P*)^2^] where *P* = (*F*_o_^2^ + 2*F*_c_^2^)/3
*S* = 0.94	(Δ/σ)_max_ < 0.001
4009 reflections	Δρ_max_ = 0.32 e Å^−^^3^
257 parameters	Δρ_min_ = −0.19 e Å^−^^3^
1 restraint	Absolute structure: Flack (1983), 1943 Friedel pairs
Primary atom site location: structure-invariant direct methods	Flack parameter: 0.15 (10)

### Special details

**Table:**

Geometry. Bond distances, angles *etc*. have been calculated using the rounded fractional coordinates. All su's are estimated from the variances of the (full) variance-covariance matrix. The cell e.s.d.'s are taken into account in the estimation of distances, angles and torsion angles
Refinement. Refinement on *F*^2^ for ALL reflections except those flagged by the user for potential systematic errors. Weighted *R*-factors *wR* and all goodnesses of fit *S* are based on *F*^2^, conventional *R*-factors *R* are based on *F*, with *F* set to zero for negative *F*^2^. The observed criterion of *F*^2^ > σ(*F*^2^) is used only for calculating -*R*-factor-obs *etc*. and is not relevant to the choice of reflections for refinement. *R*-factors based on *F*^2^ are statistically about twice as large as those based on *F*, and *R*-factors based on ALL data will be even larger.

### Fractional atomic coordinates and isotropic or equivalent isotropic displacement parameters (Å^2^)

**Table:**

	*x*	*y*	*z*	*U*_iso_*/*U*_eq_	
Cl1	0.68230 (6)	0.6442 (5)	0.97181 (6)	0.0916 (6)	
Cl2	0.50757 (6)	0.7463 (3)	0.81738 (5)	0.0709 (4)	
N1	0.56446 (17)	0.4177 (12)	0.97981 (17)	0.0530 (12)	
C1	0.6040 (3)	0.5681 (15)	0.9491 (2)	0.0630 (19)	
C2	0.5891 (2)	0.6763 (11)	0.89752 (18)	0.0550 (16)	
C3	0.5272 (2)	0.6141 (12)	0.88079 (17)	0.0490 (14)	
C4	0.4813 (3)	0.4506 (11)	0.9111 (3)	0.0510 (19)	
C5	0.4158 (2)	0.3859 (13)	0.8958 (2)	0.0593 (17)	
C6	0.3744 (2)	0.2192 (11)	0.9295 (2)	0.0647 (17)	
C7	0.3943 (2)	0.1131 (13)	0.9795 (2)	0.0623 (19)	
C8	0.4577 (2)	0.1780 (10)	0.99705 (18)	0.0553 (17)	
C9	0.50171 (19)	0.3483 (10)	0.96177 (17)	0.0477 (14)	
C10	0.3437 (3)	−0.0425 (13)	1.0129 (2)	0.0640 (19)	
C11	0.4804 (3)	0.0815 (14)	1.0504 (2)	0.074 (2)	
Cl3	0.56576 (6)	1.1324 (5)	0.69164 (6)	0.0964 (6)	
Cl4	0.73974 (6)	1.2634 (3)	0.84687 (5)	0.0694 (4)	
N2	0.6857 (2)	0.9027 (12)	0.68457 (18)	0.0627 (17)	
C12	0.6456 (3)	1.0586 (13)	0.7160 (2)	0.0540 (17)	
C13	0.6582 (2)	1.1805 (11)	0.76636 (19)	0.0553 (16)	
C14	0.7208 (2)	1.1269 (12)	0.78476 (17)	0.0500 (16)	
C15	0.7689 (2)	0.9656 (10)	0.7534 (3)	0.0420 (18)	
C16	0.8335 (3)	0.8998 (14)	0.7678 (2)	0.0620 (17)	
C17	0.8766 (2)	0.7485 (11)	0.73560 (19)	0.0590 (17)	
C18	0.8568 (2)	0.6444 (12)	0.6858 (2)	0.0610 (19)	
C19	0.7928 (2)	0.6889 (10)	0.66826 (18)	0.0567 (17)	
C20	0.74761 (19)	0.8565 (11)	0.70266 (17)	0.0507 (16)	
C21	0.9046 (4)	0.4700 (17)	0.6447 (4)	0.112 (4)	
C22	0.7674 (3)	0.5740 (13)	0.6163 (2)	0.0650 (19)	
H2	0.62010	0.78320	0.87660	0.0660*	
H5	0.40070	0.45540	0.86320	0.0710*	
H6	0.33150	0.17480	0.91870	0.0780*	
H10A	0.32250	0.12660	1.03360	0.0960*	
H10B	0.36400	−0.20380	1.03550	0.0960*	
H10C	0.31150	−0.15180	0.99130	0.0960*	
H11A	0.46530	0.24420	1.07530	0.1120*	
H11B	0.52760	0.07210	1.05100	0.1120*	
H11C	0.46280	−0.13390	1.05930	0.1120*	
H13	0.62630	1.29030	0.78600	0.0660*	
H16	0.84760	0.96260	0.80100	0.0740*	
H17	0.91970	0.71310	0.74650	0.0700*	
H21A	0.90020	0.57790	0.61130	0.1670*	
H21B	0.89310	0.23810	0.64140	0.1670*	
H21C	0.94930	0.48880	0.65650	0.1670*	
H22A	0.79460	0.66330	0.58900	0.0970*	
H22B	0.72290	0.65090	0.61180	0.0970*	
H22C	0.76820	0.33420	0.61490	0.0970*	

### Atomic displacement parameters (Å^2^)

**Table:**

	*U*^11^	*U*^22^	*U*^33^	*U*^12^	*U*^13^	*U*^23^
Cl1	0.0611 (8)	0.1339 (13)	0.0798 (10)	−0.0212 (8)	−0.0161 (7)	0.0013 (12)
Cl2	0.0827 (8)	0.0806 (7)	0.0495 (6)	0.0103 (6)	−0.0061 (6)	0.0020 (6)
N1	0.047 (2)	0.068 (2)	0.044 (2)	0.004 (2)	−0.0064 (19)	−0.008 (2)
C1	0.048 (3)	0.069 (3)	0.072 (4)	−0.003 (3)	−0.001 (3)	−0.013 (3)
C2	0.062 (3)	0.056 (3)	0.047 (2)	−0.002 (2)	0.005 (2)	−0.007 (2)
C3	0.060 (3)	0.048 (2)	0.039 (2)	0.006 (2)	0.000 (2)	−0.005 (2)
C4	0.052 (3)	0.041 (3)	0.060 (4)	0.009 (2)	−0.008 (3)	−0.020 (2)
C5	0.045 (3)	0.075 (3)	0.058 (3)	0.010 (3)	−0.003 (2)	−0.019 (3)
C6	0.046 (3)	0.067 (3)	0.081 (3)	0.007 (2)	−0.004 (2)	−0.016 (3)
C7	0.054 (3)	0.054 (3)	0.079 (4)	0.005 (3)	0.012 (3)	−0.016 (3)
C8	0.066 (3)	0.048 (3)	0.052 (3)	0.004 (2)	0.008 (2)	−0.012 (2)
C9	0.055 (2)	0.038 (2)	0.050 (3)	0.001 (2)	0.011 (2)	−0.012 (2)
C10	0.062 (4)	0.062 (3)	0.068 (3)	−0.004 (2)	0.018 (3)	−0.018 (3)
C11	0.088 (4)	0.067 (3)	0.068 (4)	0.002 (3)	−0.001 (3)	−0.004 (3)
Cl3	0.0610 (8)	0.1433 (13)	0.0848 (10)	0.0298 (9)	−0.0193 (7)	−0.0122 (13)
Cl4	0.0783 (7)	0.0777 (7)	0.0523 (6)	−0.0045 (6)	−0.0028 (6)	−0.0053 (6)
N2	0.067 (3)	0.067 (3)	0.054 (3)	0.006 (2)	−0.002 (2)	−0.002 (3)
C12	0.046 (3)	0.073 (3)	0.043 (3)	0.005 (2)	−0.002 (2)	−0.006 (3)
C13	0.042 (2)	0.061 (3)	0.063 (3)	0.008 (2)	0.005 (2)	0.002 (2)
C14	0.058 (3)	0.047 (3)	0.045 (2)	−0.003 (2)	0.006 (2)	0.002 (2)
C15	0.034 (3)	0.039 (2)	0.053 (4)	0.0013 (16)	0.004 (3)	0.0138 (19)
C16	0.070 (3)	0.057 (3)	0.059 (3)	−0.014 (3)	−0.009 (3)	0.004 (3)
C17	0.050 (3)	0.060 (3)	0.067 (3)	−0.001 (2)	−0.004 (2)	0.006 (3)
C18	0.060 (3)	0.043 (3)	0.080 (4)	−0.001 (2)	0.016 (3)	0.018 (3)
C19	0.063 (3)	0.049 (3)	0.058 (3)	−0.008 (2)	0.007 (2)	0.011 (2)
C20	0.047 (2)	0.054 (3)	0.051 (3)	0.000 (2)	0.009 (2)	0.007 (2)
C21	0.084 (5)	0.086 (5)	0.166 (8)	0.018 (3)	0.056 (5)	0.007 (4)
C22	0.084 (4)	0.072 (3)	0.039 (3)	0.016 (3)	0.007 (3)	−0.002 (3)

### Geometric parameters (Å, °)

**Table:**

Cl1—C1	1.720 (6)	C10—H10C	0.9600
Cl2—C3	1.752 (5)	C11—H11B	0.9600
Cl3—C12	1.762 (6)	C11—H11C	0.9600
Cl4—C14	1.723 (5)	C11—H11A	0.9600
N1—C9	1.383 (5)	C12—C13	1.401 (7)
N1—C1	1.274 (7)	C13—C14	1.372 (6)
N2—C20	1.352 (6)	C14—C15	1.419 (7)
N2—C12	1.303 (7)	C15—C16	1.388 (7)
C1—C2	1.421 (7)	C15—C20	1.436 (8)
C2—C3	1.351 (6)	C16—C17	1.345 (7)
C3—C4	1.377 (8)	C17—C18	1.399 (7)
C4—C5	1.410 (7)	C18—C19	1.386 (6)
C4—C9	1.421 (8)	C18—C21	1.592 (10)
C5—C6	1.376 (7)	C19—C20	1.437 (6)
C6—C7	1.407 (7)	C19—C22	1.498 (7)
C7—C10	1.474 (7)	C13—H13	0.9300
C7—C8	1.388 (6)	C16—H16	0.9300
C8—C11	1.491 (7)	C17—H17	0.9300
C8—C9	1.441 (6)	C21—H21A	0.9600
C2—H2	0.9300	C21—H21B	0.9600
C5—H5	0.9300	C21—H21C	0.9600
C6—H6	0.9300	C22—H22A	0.9600
C10—H10A	0.9600	C22—H22B	0.9600
C10—H10B	0.9600	C22—H22C	0.9600
			
Cl1···H22A^i^	3.0300	H5···H16^iii^	2.5500
Cl2···H5	2.7300	H6···H10C	2.3100
Cl2···H13^ii^	3.1300	H6···Cl4^iii^	3.1500
Cl2···H17^iii^	3.1400	H10A···C22^viii^	3.0400
Cl3···H21C^iv^	2.9500	H10A···H22B^viii^	2.3800
Cl4···H6^v^	3.1500	H10B···C11	2.6500
Cl4···H16	2.7600	H10B···H11C	2.1200
N1···H11B	2.4100	H10C···H6	2.3100
N2···H22B	2.2500	H11A···H11C^vi^	2.5200
C3···C4^vi^	3.558 (7)	H11B···N1	2.4100
C4···C3^ii^	3.558 (7)	H11C···H11A^ii^	2.5200
C5···C6^vi^	3.543 (7)	H11C···C10	2.7200
C6···C5^ii^	3.543 (7)	H11C···H10B	2.1200
C8···C9^ii^	3.553 (6)	H13···Cl2^vi^	3.1300
C9···C8^vi^	3.553 (6)	H16···Cl4	2.7600
C14···C15^vi^	3.584 (6)	H16···H5^v^	2.5500
C15···C14^ii^	3.584 (6)	H17···H21C	2.5400
C18···C21^vi^	3.598 (9)	H17···Cl2^v^	3.1400
C19···C20^ii^	3.563 (6)	H21A···C22	2.7000
C20···C19^vi^	3.563 (6)	H21A···H22A	2.2400
C21···C18^ii^	3.598 (9)	H21B···C18^ii^	2.7300
C10···H11C	2.7200	H21B···C19^ii^	3.0700
C11···H10B	2.6500	H21B···C21^ii^	3.0800
C18···H21B^vi^	2.7300	H21B···C22	2.9500
C19···H21B^vi^	3.0700	H21C···H17	2.5400
C19···H22C^vi^	2.9600	H21C···Cl3^ix^	2.9500
C20···H22C^vi^	2.9800	H22A···C21	2.7600
C21···H21B^vi^	3.0800	H22A···H21A	2.2400
C21···H22C	2.9200	H22A···Cl1^x^	3.0300
C21···H22A	2.7600	H22B···N2	2.2500
C22···H22C^vi^	3.0400	H22B···H10A^vii^	2.3800
C22···H10A^vii^	3.0400	H22C···C19^ii^	2.9600
C22···H21B	2.9500	H22C···C20^ii^	2.9800
C22···H21A	2.7000	H22C···C21	2.9200
H5···Cl2	2.7300	H22C···C22^ii^	3.0400
			
C1—N1—C9	118.0 (4)	H11A—C11—H11B	110.00
C12—N2—C20	115.8 (5)	Cl3—C12—N2	115.9 (4)
Cl1—C1—N1	117.3 (4)	Cl3—C12—C13	115.8 (4)
N1—C1—C2	125.6 (5)	N2—C12—C13	128.3 (5)
Cl1—C1—C2	117.2 (4)	C12—C13—C14	115.5 (4)
C1—C2—C3	115.9 (4)	Cl4—C14—C13	118.3 (3)
Cl2—C3—C2	116.7 (3)	Cl4—C14—C15	120.8 (4)
C2—C3—C4	122.6 (5)	C13—C14—C15	121.0 (4)
Cl2—C3—C4	120.7 (4)	C14—C15—C16	125.9 (6)
C3—C4—C5	124.7 (6)	C14—C15—C20	116.2 (4)
C5—C4—C9	118.4 (5)	C16—C15—C20	117.8 (5)
C3—C4—C9	116.9 (5)	C15—C16—C17	122.5 (5)
C4—C5—C6	119.4 (5)	C16—C17—C18	120.3 (4)
C5—C6—C7	122.7 (4)	C17—C18—C19	121.7 (4)
C6—C7—C8	120.3 (4)	C17—C18—C21	123.8 (5)
C6—C7—C10	117.0 (4)	C19—C18—C21	114.5 (5)
C8—C7—C10	122.6 (5)	C18—C19—C20	117.4 (4)
C7—C8—C9	117.5 (4)	C18—C19—C22	124.8 (4)
C7—C8—C11	122.3 (4)	C20—C19—C22	117.8 (4)
C9—C8—C11	120.2 (4)	N2—C20—C15	123.2 (4)
N1—C9—C8	117.2 (4)	N2—C20—C19	116.6 (4)
C4—C9—C8	121.8 (4)	C15—C20—C19	120.2 (4)
N1—C9—C4	121.0 (4)	C12—C13—H13	122.00
C3—C2—H2	122.00	C14—C13—H13	122.00
C1—C2—H2	122.00	C15—C16—H16	119.00
C4—C5—H5	120.00	C17—C16—H16	119.00
C6—C5—H5	120.00	C16—C17—H17	120.00
C7—C6—H6	119.00	C18—C17—H17	120.00
C5—C6—H6	119.00	C18—C21—H21A	109.00
C7—C10—H10A	110.00	C18—C21—H21B	109.00
C7—C10—H10B	109.00	C18—C21—H21C	110.00
H10A—C10—H10B	110.00	H21A—C21—H21B	109.00
H10A—C10—H10C	109.00	H21A—C21—H21C	109.00
C7—C10—H10C	109.00	H21B—C21—H21C	109.00
H10B—C10—H10C	109.00	C19—C22—H22A	109.00
C8—C11—H11B	109.00	C19—C22—H22B	109.00
C8—C11—H11C	110.00	C19—C22—H22C	110.00
C8—C11—H11A	109.00	H22A—C22—H22B	110.00
H11A—C11—H11C	109.00	H22A—C22—H22C	110.00
H11B—C11—H11C	109.00	H22B—C22—H22C	109.00
			
C9—N1—C1—Cl1	−178.7 (4)	C7—C8—C9—C4	0.7 (6)
C9—N1—C1—C2	0.8 (8)	C11—C8—C9—N1	−0.4 (6)
C1—N1—C9—C4	−1.9 (7)	C11—C8—C9—C4	−178.6 (4)
C1—N1—C9—C8	180.0 (5)	C7—C8—C9—N1	178.9 (4)
C20—N2—C12—Cl3	177.8 (4)	Cl3—C12—C13—C14	−179.0 (4)
C20—N2—C12—C13	−0.9 (8)	N2—C12—C13—C14	−0.3 (8)
C12—N2—C20—C15	0.8 (7)	C12—C13—C14—Cl4	−178.9 (4)
C12—N2—C20—C19	−179.2 (4)	C12—C13—C14—C15	1.5 (7)
N1—C1—C2—C3	0.7 (8)	Cl4—C14—C15—C16	0.2 (7)
Cl1—C1—C2—C3	−179.8 (4)	Cl4—C14—C15—C20	178.9 (3)
C1—C2—C3—Cl2	179.8 (4)	C13—C14—C15—C16	179.7 (5)
C1—C2—C3—C4	−1.2 (7)	C13—C14—C15—C20	−1.6 (7)
Cl2—C3—C4—C5	−2.4 (7)	C14—C15—C16—C17	−179.3 (5)
Cl2—C3—C4—C9	179.2 (3)	C20—C15—C16—C17	2.0 (8)
C2—C3—C4—C9	0.2 (7)	C14—C15—C20—N2	0.3 (7)
C2—C3—C4—C5	178.7 (5)	C14—C15—C20—C19	−179.6 (4)
C3—C4—C5—C6	179.9 (5)	C16—C15—C20—N2	179.2 (5)
C9—C4—C5—C6	−1.7 (7)	C16—C15—C20—C19	−0.8 (7)
C5—C4—C9—C8	0.9 (7)	C15—C16—C17—C18	−1.3 (8)
C3—C4—C9—N1	1.4 (7)	C16—C17—C18—C19	−0.8 (7)
C3—C4—C9—C8	179.4 (4)	C16—C17—C18—C21	178.8 (5)
C5—C4—C9—N1	−177.2 (4)	C17—C18—C19—C20	1.9 (7)
C4—C5—C6—C7	1.0 (7)	C17—C18—C19—C22	−177.8 (4)
C5—C6—C7—C10	176.0 (5)	C21—C18—C19—C20	−177.7 (4)
C5—C6—C7—C8	0.7 (7)	C21—C18—C19—C22	2.6 (7)
C6—C7—C8—C9	−1.5 (7)	C18—C19—C20—N2	178.9 (4)
C6—C7—C8—C11	177.8 (4)	C18—C19—C20—C15	−1.1 (6)
C10—C7—C8—C9	−176.5 (4)	C22—C19—C20—N2	−1.3 (6)
C10—C7—C8—C11	2.8 (7)	C22—C19—C20—C15	178.7 (4)

Symmetry codes: (i) −*x*+3/2, *y*, *z*+1/2; (ii) *x*, *y*−1, *z*; (iii) *x*−1/2, −*y*+1, *z*; (iv) *x*−1/2, −*y*+2, *z*; (v) *x*+1/2, −*y*+1, *z*; (vi) *x*, *y*+1, *z*; (vii) −*x*+1, −*y*+1, *z*−1/2; (viii) −*x*+1, −*y*+1, *z*+1/2; (ix) *x*+1/2, −*y*+2, *z*; (x) −*x*+3/2, *y*, *z*−1/2.

### Hydrogen-bond geometry (Å, °)

**Table:**

*D*—H···*A*	*D*—H	H···*A*	*D*···*A*	*D*—H···*A*
C5—H5···Cl2	0.93	2.73	3.094 (5)	104
C11—H11B···N1	0.96	2.41	2.825 (7)	106
C16—H16···Cl4	0.93	2.76	3.135 (6)	105
C22—H22B···N2	0.96	2.25	2.744 (7)	111
